# Distribution of dense breasts using screening mammography in Korean women: a retrospective observational study

**DOI:** 10.4178/epih/e2014027

**Published:** 2014-11-04

**Authors:** Jong-Myon Bae, Sang Yop Shin, Eun Hee Kim, Yoon-Nam Kim, Chung Mo Nam

**Affiliations:** 1 Department of Preventive Medicine, Jeju National University School of Medicine, Jeju, Korea; 2 Korea Medical Institute, Seoul, Korea; 3 Severance Hospital, Seoul, Korea; 4 Department of Preventive Medicine, Yonsei University College of Medicine, Seoul, Korea

**Keywords:** Breast neoplasms, Early detection of cancer, Mass screening, Mammography

## Abstract

**OBJECTIVES:**

This retrospective observational study evaluated the distribution of dense breasts by age group among healthy Korean women.

**METHODS:**

Participants were women aged 30 years and older who voluntarily underwent screening mammography between January 2007 and December 2011. Women who received the Breast Imaging Reporting and Data System for mammographic density of 3 or 4 were defined as having dense breasts. The proportion of women with dense breasts (PDB, %) was calculated by dividing the number of participants with dense breasts by the total number of participants.

**RESULTS:**

Among the 231,058 women who participated, 78.15% were classified as having dense breasts. PDB was highest in the youngest age group (PDB=94.87%) and lowest in the oldest age group. The greatest difference in PDB between adjacent age groups was observed in the group aged 60-64 years.

**CONCLUSIONS:**

The results show that the proportion of dense breasts by age group increased in all age groups, except in those aged 35-39 years. These findings suggest an association between the age distribution of dense breasts and trends in breast cancer incidence. Further studies are needed to estimate the change in breast cancer incidence rate by age and the accumulation of fatty breast tissue in Korean women.

## INTRODUCTION

Breast cancer is the second most common type of cancer among Korean women [1] and the most common among women globally [2]. The incidence of breast cancer is lower among women in Asia than among women in Western countries [3], with different trends in age at diagnosis [4]. These epidemiological differences may be due to biological variation in estrogen receptor (ER) status among racial/ethnic groups [5] and the prevalence of risk factors such as mammographic breast density [6].

Breast density has been shown to be associated with reproductive and hormonal factors [7], and among Western women, breast density is considered one of the strongest known risk factors for breast cancer development [8]. Breast density also varies among racial groups and may explain some of the racial disparity in breast cancer incidence [9].

Mammographic density and the age-specific incidence of breast cancer differ markedly between women in Asian and Western countries [10-13]. Therefore, there may be differences in the relationship between breast density and breast cancer risk in Asian women [9], including Korean women [14,15], compared to women in Western countries.

Evaluating the distribution of breast density by age group is necessary to determine whether there is an effect of breast density on the risk of breast cancer [16]. Two studies have examined breast density among healthy Korean women [15,17], although these data were collected in 2002 [15] and 1998 [17]. Because the incidence rate of breast cancer has increased in Asian countries, including South Korea [1,11], the aim of the present study was to describe the distribution of mammographic breast density by age group in a Korean population.

## MATERIALS AND METHODS

### Study participants

Study participants were recruited through cancer screening programs conducted by the Korea Medical Institute (KMI), as described in Bae et al. [18]. The KMI is a non-profit foundation that has specialized in comprehensive health examination services since 1985. In this screening program, digital mammography has been the primary modality for both initial and follow-up screenings for breast cancer since 2005.

Study participants were Korean women aged 30 years and older who voluntarily underwent screening mammography between January 2007 and December 2011. Women who reported a prior history of breast cancer on a structured questionnaire were excluded because the mammography was a follow-up, rather than a screening test. Participant age was defined as the age at which the screening mammogram was received.

### Definition of dense breasts

For each participant, breast density was assigned one of four Breast Imaging Reporting and Data System (BI-RADS) categories. Dense breasts were defined as category 3 (heterogeneously dense [51-75% glandular]) or category 4 (extremely dense [>75% glandular]) [19].

All participants consented to screening mammography and the use of personal data for research. This study protocol was approved by the institutional review board of Jeju National University Hospital (No. 2013-09-005).

### Statistical analysis

Participants were categorized by five-year age intervals (30-34, 35-39, and so on, up to 80-84 years, and ≥85 years). The proportion of participants with dense breasts (PDB, %) was calculated by dividing the number of women with dense breasts by the total number of participants. The dropping PDB (%) was determined by the difference in PDB between adjacent age groups. We evaluated the difference in the proportion of dense breasts by comparing each age group with the equivalent age group of the data published by Kim et al. [17]. These data, as opposed to those published by Cho et al. [15], were used because more detailed information was available in the former publication. The subjects of Kim et al. [17] were volunteers of screening mammography at a medical institute in 1998. A chi-squared test was used to analyze differences in PDB by age group between the KMI database and the dataset of Kim et al. [17]. A *p*-value of less than 0.05 was considered statistically significant. All statistical tests were performed using STATA version 12 (StataCorp, College Station, TX, USA).

## RESULTS

The distribution of PDB by age group is shown in Table 1. In total, 231,058 participants were enrolled in the study, and the overall PDB was 78.15%. The highest PDB was observed in the youngest age group (30-34 years, 94.87%). PDB decreased as age increased. The greatest difference in PDB was observed between the 55-59 years and 60-64 years age group (-20.04%).

**Figure 1.**
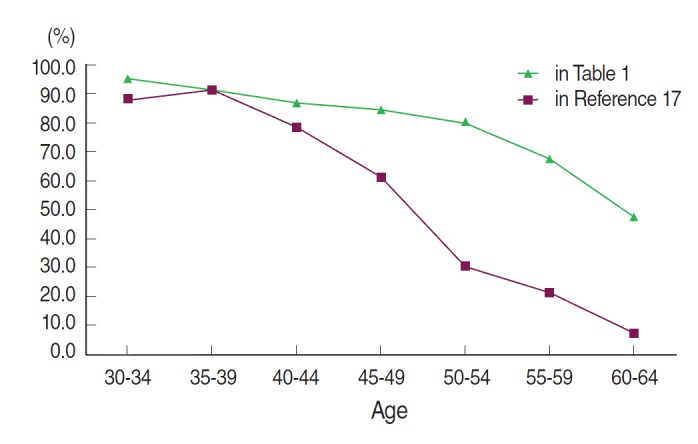
Curves of proportion of dense breast by age groups.

Figure 1 and Table 2 illustrate the difference in PDB distribution between the KMI database and the dataset of Kim et al. [17], stratified by age group. There was an increase in PDB after ten years in all age groups, except women 35-39 years old. The greatest increases were observed among the age groups of women older than 50 years. The youngest age group with a PDB less than 50% in Kim et al. [17] was 50-54 years, which shifted to the 60-64 age group after a 10-year period.

## DISCUSSION

Our results show that the distribution of dense breasts by age group has changed over a ten-year period, with an increase in PDB observed for all age groups, except those aged 35-39 years. These results suggest that the different patterns in age at diagnosis of breast cancer between Asian and Western women may be related to the difference in PDB. It is also possible that changes in the PDB could be associated with the rising incidence of breast cancer.

Our study had three main limitations. The first issue is the possibility of self-selection bias, a type of selection bias. Because the study participants voluntarily participated in the screenings, they are not representative of all Korean women. However, the aim of this study was not to evaluate any benefit from the screening intervention, but rather to describe the distribution of dense breasts. Since the participants did not know whether they had dense breasts as the time of the screening, they are likely to be representative of women who would volunteer for a screening mammography study. The second issue is the reliability of mammographic density determination. PDB was determined using the BI-RADS four-category scoring system for mammographic density [19], not the BI-RADS five-category scoring system for recommendataion. By the way, Kerlikowske et al. [20] reported that the kappa value in breast density was highest (0.81) among BI-RADS parameters such as lesion finding, assessment category and recommendation. The last limitation is the comparability of our results with those in Kim et al. [17]. The source population in both studies was healthy women who underwent screening mammography at a medical institute, and both studies used the BI-RADS reporting system [19]. However, while comparable trends in PDB between the two studies can be discussed, a direct comparison of PDB is not possible due to the differences in the medical setting and the mammographic machine used.

The phenomenon of rapidly increasing incidence rates of breast cancer among Asian women could be explained by the combination of a diet rich in saturated fat and a sedentary lifestyle, as well as early menarche, decreased parity, and delayed childbearing, commonly referred to as the Westernization of lifestyle [21]. Shin et al. [11] and Sehn et al. [21] have argued that the changing trends in breast cancer incidence among Asian women are due to a birth cohort effect that corresponds to the adoption of a Western lifestyle. However, data published by Bae et al. showing incidence trends in Korean women over a ten-year period [4] cast doubt on the birth cohort effect hypothesis. In addition, Westernization of lifestyle has been associated with increased breast density [15]. Thus, an age-period-cohort analysis is needed to evaluate an association between changes of distribution of dense breasts and trends in incidence rates among Asian women.

Because PDB is higher among women from Asian countries than women from Western countries [17], the accuracy of screening mammography could be reduced, leading to higher rates of interval cancers and worsening prognosis [22,23]. If dense breasts are shown to be an independent risk factor for breast cancer in Asian women [24,25], women with dense breasts could be considered to be at high risk for breast cancer development and included in high-risk screening programs [19,26]. Further cohort studies incorporating follow-up screening mammography are therefore required to evaluate the relative risk of breast cancer among women whose breasts remain dense and women whose breasts develop adipose tissue over time [27].

In conclusion, this study suggests that over a ten-year period, PDB changes with age in Korean women. This suggests an association between the age distribution of women with dense breasts and trends in the incidence rate of breast cancer. Additional studies are needed to estimate the change in incidence rate by age group and the accumulation of breast fatty tissue in Korean women.

## Figures and Tables

**Table 1.** The proportion of dense breasts (PDB) by age group^1^

Age at baseline mammography (yr)	Participants (n) [A]	Participants with dense breasts (n) [B]	PDB (%) [C]	Decrease in PDB (%) [D]
30-34	24,339	23,091	94.87	
35-39	39,460	35,848	90.85	-4.03
40-44	48,052	41,636	86.65	-4.20
45-49	39,403	33,180	84.21	-2.44
50-54	27,914	22,360	80.10	-4.10
55-59	21,049	14,177	67.35	-12.75
60-64	12,784	6,048	47.31	-20.04
65-69	7,935	2,514	31.68	-15.63
70-74	5,039	1,116	22.15	-9.54
75-79	2,885	442	15.32	-6.83
80-84	1,582	132	8.34	-6.98
85+	616	39	0.06	-2.01
Total	231,058	180,583	78.15	

1 C=B/A; D=C_i_-C_i-1_ where ‘i’ refers to an age group and ‘i-1’ refers to the immediately preceding age group.

**Table 2.** Comparison of the proportion of dense breasts (PDB): snapshot by age at the time of study of two separate study groups separated by a ten-year interval

Age at baseline mammography	PDB in this study (%)	PDB in Kim et al. [17] (%)	Difference in PDB	p-value^1^
30-34	94.87	88.10	6.8	0.005
35-39	90.85	91.14	-0.3	0.90
40-44	86.65	78.30	8.4	0.012
45-49	84.21	61.11	23.1	<0.001
50-54	80.10	30.06	50.0	<0.001
55-59	67.35	21.05	46.3	<0.001
60-64	47.31	7.04	40.3	<0.001

1 p-value using chi-squared test.
